# Correction: Effects of high-intensity functional training on physical fitness and sport-specific performance among the athletes: A systematic review with meta-analysis

**DOI:** 10.1371/journal.pone.0299281

**Published:** 2024-02-16

**Authors:** The PLOS ONE Staff

## Notice of Republication

This article was republished on January 25, 2024, to correct an error in the author list: Xinzhi Wang was erroneously displayed as the corresponding author instead of Kim Geok Soh. The publisher apologizes for the error. Please download this article again to view the correct version. The originally published, uncorrected article and the republished, corrected article are provided here for reference.

## Supporting information

S1 File Originally published, uncorrected article. (PDF)

S2 File Republished, corrected article. (PDF)
