# Supplementary material for: Correction: Effects of high-intensity functional training on physical fitness and sport-specific performance among the athletes: A systematic review with meta-analysis
Source: PLoS One. 2024 Feb 16;19(2):e0299281. doi: 10.1371/journal.pone.0299281 (PMC10871485; doi:10.1371/journal.pone.0299281)
Supplement: S2 File — (PDF) [file pone.0299281.s002.pdf]

PLOS ONE

RESEARCH ARTICLE

Effects of high-intensity functional training on physical fitness and sport-specific performance among the athletes: A systematic review with meta-analysis

Xinzhi Wang, Kim Geok Soh*, Shamsulariffin Samsudin, Nuannuan Deng, Xutao Liu, Yue Zhao, Saddam Akbar

Faculty of Educational Studies, Department of Sports Studies, Universiti Putra Malaysia, Serdang, Selangor, Malaysia

* kims@upm.edu.my

Check for updates

Abstract

Objective

This study aims to meta-analyze the impact of high-intensity functional training on athletes' physical fitness and sport-specific performance.

Methods

A systematic search was conducted in five well-known academic databases (PubMed, Scopus, Web of Science, EBSCOhost, and the Cochrane Library) up to July 1, 2023. The literature screening criteria included: (1) studies involving healthy athletes, (2) a HIFT program, (3) an assessment of outcomes related to athletes' physical fitness or sport-specific performance, and (4) the inclusion of randomized controlled trials. The Physical Therapy Evidence Database (PEDro) scale was used to evaluate the quality of studies included in the meta-analysis.

Results

13 medium- and high-quality studies met the inclusion criteria for the systematic review, involving 478 athletes aged between 10 and 24.5 years. The training showed a small to large effect size (ES = 0.414–3.351; all $p < 0.05$) in improving upper and lower body muscle strength, power, flexibility, and sport-specific performance.

Conclusion

High-intensity functional training effectively improves athletes' muscle strength, power, flexibility, and sport-specific performance but has no significant impact on endurance and agility. Future research is needed to explore the impact of high-intensity functional training on athletes' speed, balance, and technical and tactical performance parameters.

OPEN ACCESS

Citation: Wang X, Soh KG, Samsudin S, Deng N, Liu X, Zhao Y, et al. (2023) Effects of high-intensity functional training on physical fitness and sport-specific performance among the athletes: A systematic review with meta-analysis. PLoS ONE 18(12): e0295531. https://doi.org/10.1371/journal.pone.0295531

Editor: Jovan Gardasevic, University of Montenegro, MONTENEGRO

Received: October 26, 2023

Accepted: November 19, 2023

Published: December 8, 2023

Peer Review History: PLOS recognizes the benefits of transparency in the peer review process; therefore, we enable the publication of all of the content of peer review and author responses alongside final, published articles. The editorial history of this article is available here: https://doi.org/10.1371/journal.pone.0295531

Copyright: © 2023 Wang et al. This is an open access article distributed under the terms of the Creative Commons Attribution License, which permits unrestricted use, distribution, and reproduction in any medium, provided the original author and source are credited.

Data Availability Statement: All relevant data are within the paper and its Supporting information files.

Funding: The author(s) received no specific funding for this work.

Competing interests: The authors have declared that no competing interests exist.

1 Introduction

High-intensity Functional Training (HIFT) is a cutting-edge training method endorsed by CrossFit [1]. Its primary aim is to generate substantial metabolic demands through rapid and repetitive training with minimal or no rest. Over recent years, this high-intensity training approach has garnered widespread popularity among athletes and the general population globally, owing to its brief duration and effectiveness [2–7]. HIFT is characterized by its emphasis on multi-joint, functional, explosive, and continuous full-body movements within various training programs, combining strength exercises with aerobic and bodyweight exercises [8,9]. Its objective is to optimize athletes' movement efficiency within relatively short periods [10,11]. Unlike traditional training methods that focus on isolated muscle groups and specific movement patterns, HIFT seeks to replicate real-life activities and sports demands by incorporating a wide range of functional movements performed at a high intensity [12].

Physical fitness and sport-specific performance determine athletes' competitive abilities [13]. Specialized sports performance refers to athletes' skill level and effectiveness in specific sports processes. Physical fitness forms the foundation for specialized sports performance, with muscle strength, endurance, and agility positively influencing athletes' movement speed. For example, Chaabene et al. found that upper and lower body muscle strength positively impacted boxers' ability to deliver rapid punches. Basketball players require robust aerobic endurance to sustain performance throughout the game [14], and excellent quadriceps muscle strength can optimize hiking performance [15]. The mobility of the hip joint is crucial for taekwondo athletes' kicking performance. Additionally, Pan et al.'s research revealed that single-handed dinghy competition performance is influenced by athletes' muscle strength, flexibility, power, muscular endurance, and cardiovascular fitness [16]. Developing physical attributes such as muscle strength, power, and endurance is crucial for athletes to achieve outstanding performance in competitions [17,18].

Sport-specific performance is a significant prerequisite for athletes to excel in sport-specific scenarios [19,20]. Scientific training methods enhance athlete performance [21]. A singular traditional training approach may isolate Kinematic chains during training, thereby reducing the effectiveness of improving athletes' specific sports performance [17,22]. Dr. Arthur Steidler drew inspiration from the Kinematic chain theory of mechanical engineer Franz Reuleaux and categorized the human body's motion into the upper limb chain, core chain, and lower limb chain [23]. These chains are interconnected, forming a cohesive unit where force generated in one link is transmitted continuously to the link, following a specific sequence of force generation. This sequence transfers the force along the joints to the distal links, enabling optimal motion performance [24]. HIFT emphasizes functional multi-joint activities that engage multiple muscle groups, joints, planes, and dimensions [25]. Research has shown that the functional multi-joint activities in HIFT effectively enhance general physical fitness parameters and athletic performance in diverse training populations [10,26–29].

HIFT suits individuals with diverse training levels [30]. HIFT is a versatile training modality characterized by constantly varying functional movements [31]. It encompasses high-intensity, short-term, and ever-changing functional training patterns involving whole-body, multi-plane movements [32]. These patterns consist of various exercises with different types and durations, occasionally performed without intervals [33,34]. This training method can be customized in intensity and training patterns to accommodate individuals with varying physical conditions [35–37]. Several studies have investigated the effects of HIFT on various populations, including military personnel [5], firefighters [38], football players [39], recreational runners [40], wrestlers [41], and adolescents [42]. Wilke and Mohr conducted a systematic review with a multilevel meta-analysis [30]. They identified 16 studies that met their inclusion criteria,

although the included studies focused primarily on recreationally active and inactive adults, with limited research on athletes [30].

Glassman claims that HIFT can stimulate neuroendocrine responses, increase testosterone, insulin, and growth hormone, and improve exercise performance [43]. By integrating cardiovascular, neuromotor, and muscle functions through aerobic and anaerobic exercise strategies, HIFT elicits greater muscle recruitment than repetitive high-intensity interval training (HIIT) [30,44]. It develops and enhances muscle strength, power, hypertrophy, and aerobic endurance [27,45,46]. Additionally, HIFT can be tailored to specific sports, training patterns, and athletes' needs [37]. HIFT-based workouts like CrossFit, Fran, and Cindy have gained popularity in fitness communities worldwide [31,47]. As a result, many athletes and coaches have integrated HIFT into their training regimens, aiming to gain a competitive edge.

Given the importance of understanding the impact of HIFT on athletes, conducting a comprehensive systematic review in this area is crucial. Therefore, this systematic review aims to thoroughly evaluate the current body of literature on the effects of HIFT on athletes. Additionally, we aim to critically assess the impact of HIFT on various aspects of athletic performance, such as strength, power, speed, agility, endurance, flexibility, and overall athletic performance in different sports events, through meta-analysis. The findings of this review will provide coaches and athletes with low-cost and efficient training plans in sports training. By examining the available evidence and synthesizing the results, valuable insights into the effectiveness of HIFT as a training method for athletes can be obtained, along with identifying potential areas for future research.

2 Methods

2.1 Protocol and registration

This Systematic review was based on the Preferred Reporting Items for Systematic Reviews and Meta-Analyses (PRISMA) guidelines and registered on the International Prospective System Review Register (PROSPERO) website. The registration number is CRD4202343705.

2.2 Search strategy

A systematic search of relevant literature was conducted from publication to July 2023 using five renowned academic databases: PubMed, Scopus, Web of Science, EBSCOhost, and Cochrane Library. The search focused on titles, keywords, and abstracts. The main keywords used in retrieving relevant literature on the impact of high-intensity functional training on athletes were: ("high intensity") AND ("functional training" OR "functional exercise" OR "functional workout" OR "power training" OR "CrossFit" OR "extreme conditioning program") AND ("athletes" OR "professional athletes" OR "elite athletes" OR "college athletes" OR "players"). In addition, we manually searched and reviewed potentially relevant literature through Google Scholar and reviewed the article's reference list.

2.3 Eligibility criteria

Table 1 summarizes the inclusion criteria based on the PICOS method used in this study. The PICOS method includes population, intervention, comparison, outcomes, and study design [48]. Therefore, the selected research should meet the following criteria: (1) Full-text peer-reviewed journal articles published in English that describe the impact of high-intensity functional training interventions on athletes; (2) Studies conducted on healthy athletes, regardless of gender or age; (3) Evaluation of at least one outcome related to the physical fitness or performance of athletes; (4) The included studies must be randomized controlled trials.

Table 1. PICOS eligibility inclusion criteria.

Category	Inclusion Criteria
Population	Healthy athletes
Intervention	High intensity Functional training
Comparative intervention	Two or multiple groups of controlled trials
Outcome	At least one measure related to physical fitness or sports-specific performance
Study design	Randomized controlled trial

https://doi.org/10.1371/journal.pone.0295531.t001

2.4 Study selection

The filtering results from all databases were imported into Endnote x9 software. After removing duplicates, two reviewers (WX and RW) independently screened all English journals based on titles, keywords, or abstracts to assess whether these articles met the inclusion criteria. Subsequently, the reviewers conducted a full-text evaluation of the remaining research. Any discrepancies in the selection between the two reviewers were resolved through consultation with a third reviewer (ZY). The two reviewers extracted data from papers that matched the inclusion criteria (WX and RW), and the final data were reviewed by the third reviewer (ZY). The screening process is illustrated in Fig 1.

2.5 Data extraction and synthesis

The following data were extracted based on the inclusion criteria: (1) Author and year of publication; (2) Sample size; (3) Characteristics of the participants (such as gender, age, and level of exercise); (4) Intervention characteristics (type, frequency, and duration); (5) Study outcomes. The mean and standard deviation of the included studies were summarized. If the mean and standard deviation of intervention results were not reported in the articles, other data were extracted and converted to mean and standard deviation using established methods [49–52]. The corresponding author was contacted if relevant data were unavailable in the articles.

2.6 Study quality assessment

The Physiotherapy Evidence Database (PEDro) scale, a reliable tool for evaluating the quality of experimental research methodology, was used for study quality assessment [53,54]. The PEDro scale consists of 11 items evaluating randomization, blinding, group comparison, and data analysis [53]. Independent reviewers assessed the quality of articles using a "yes" (1) or "no" (0) rating for each item. The scale ranges from 0 to 10, with higher scores indicating better methodological quality of the article. Articles with a PEDro score of five or higher were deemed to have high methodological quality, while those scoring below five were considered to have poor methodological quality [54]. The quality evaluation and scoring process using the PEDro scale involved two independent reviewers, and any disagreements were settled through discussion with the third reviewer (Table 2).

2.7 Statistical analysis

The Comprehensive Meta-Analysis software was used to analyse the data in this systematic review (CMA; Biostat et al., Version 3). The random-effects model was employed for meta-analysis [55], and effect sizes (ES; Hedge's g) were calculated using mean and standard deviation. The interpretation of effect size was categorized as follows: trivial effect size (<0.2), small effect size (0.20–0.6), medium effect size (0.6–1.2), large effect size (1.2–2.0), very large effect size (2.0–4.0), and extremely large effect size (>4.0) [56]. The I^2 statistic was used to evaluate

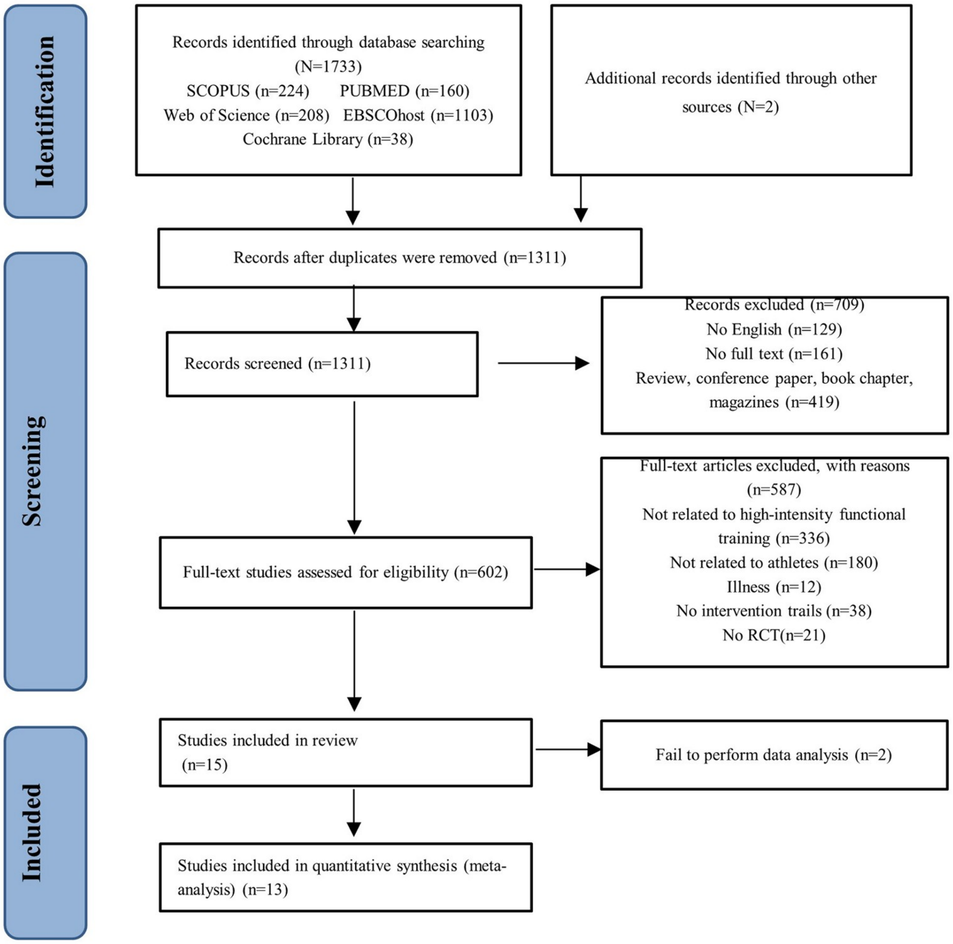

Fig 1. Flow chart of PRISMA article screening.

https://doi.org/10.1371/journal.pone.0295531.g001

the heterogeneity of the included studies, with values less than 50% indicating low heterogeneity, 50–75% indicating moderate heterogeneity, and greater than 75% indicating high heterogeneity [57]. The Chi-squared test was used to evaluate the statistical significance of heterogeneity, with $p<0.05$ considered significant. Egger's test was employed to assess publication bias; sensitivity analyses were performed to investigate sources of heterogeneity by removing one study at a time, and the stability of the overall findings was assessed by excluding individual studies [58].

3 Results

3.1 Study selection

The process of literature screening is outlined in Fig 1. 1,735 articles were initially identified from electronic databases, including 224 from Scopus, 160 from PubMed, 208 from Web of Science, 1,103 from EBSCOhost, and 38 from Cochrane Library (reference = 2). After removing duplicates using Endnote software, 1,311 articles remained for further screening. Among these, 129 were non-English articles, 161 needed full text, and 419 were not journal articles. After screening based on title and abstract, 36 articles were identified as potentially eligible and

Table 2. Methodological quality assessment for inclusion studies.

Study	Eligibility Criteria	Random Allocation	Allocation Concealment	Baseline Comparability	Blind Participants	Blind Therapist	Blind Assessor	Follow-Up	Intention to Treat Analysis	Between Group Comparisons	Point Measure and Variability	Total PEDro Score
Osipov et al. 2019 [67]	1	1	0	0	0	0	0	1	0	1	1	5
Yüksel et al. 2019 [70]	0	1	0	1	0	0	0	1	0	1	1	5
Ambroży et al. 2022 [59]	1	1	0	1	0	0	0	1	0	1	1	6
Kudryavtsev et al. 2023 [63]	1	1	0	1	0	0	0	1	0	1	1	6
Zhu. 2023 [69]	1	1	0	0	0	0	0	1	0	1	1	5
Osipov et al. 2022 [65]	1	1	0	1	0	0	0	1	0	1	1	6
Galimova et al. 2018 [62]	1	1	0	1	0	0	0	1	0	1	1	6
Türker & Yüksel. 2020 [68]	1	1	0	0	0	0	0	1	0	1	1	5
Mischenko et al. 2021 [64]	1	1	0	1	0	0	0	1	0	1	1	6
Caloglu & Yuksel. 2020 [41]	0	1	0	1	0	0	0	1	0	1	1	5
Avetisyan et al. 2022 [60]	1	1	0	1	0	0	0	1	0	1	1	6
Bozdoğan. 2021 [61]	1	1	0	1	0	0	0	1	0	1	1	6
Osipov et al. 2017 [66]	1	1	0	0	0	0	0	1	0	1	1	5

https://doi.org/10.1371/journal.pone.0295531.t002

underwent full-text evaluation. Of these, 336 articles were unrelated to the subject area, 192 were unrelated to healthy athletes, 38 did not involve experimental intervention, and 21 were not randomized controlled trials, resulting in their exclusion. Two articles needed to provide more information for inclusion in the meta-analysis and were also excluded. Finally, 13 articles met the inclusion criteria and were included in the meta-analysis.

3.2 Study quality assessment

Detailed information on the quality scores of each included study's PEDro scale is presented in Table 2. The PEDro scale scores ranged from 5 to 6. Seven studies scored 6 points [59–65], while six studies scored 5 points [41,66–70]. These scores indicate that the included articles are of good quality. However, none of the studies met the criteria for allocation concealment, blinding of participants, therapists, and assessors as assessed by the PEDro scale. Two studies did not specify the eligibility criteria for participant inclusion [41,70].

3.3 Participant characteristics

Table 3 summarizes the participant characteristics of 13 studies that met the inclusion criteria, and the content included is shown below. (1) Athlete classification: among the 13 articles, there was one article about aerobic gymnasts [69], one article about volleyball players [61], and 11 articles about martial arts athletes [41,59,60,63–68,70]. (2) Gender classification: Eight out of 13 articles investigated male athletes [40,41,59,60,63,65,67,68,70,71], and two articles investigated female athletes [61,64]. However, three studies did not report gender information [62,66,69]. (3) Sample size: the total number of subjects collected in the 13 studies was 530, ranging from 20 to 60, with an average sample size of 35.33 [41,59–61,63–70]. The number of martial arts athletes was the highest, at 432 [41,59,60,63–68,70]. (4) Age: all studies reported the ages of the participants. The age range was between 10 and 24 years old [41,59–61,63–70]. (5) Athlete training level: three studies were on elite athletes [63,65,67], seven studies on professional sports [41,59,61,64,66,68,69], and one study each on amateur athletes [70], junior athletes [60], and college athletes [62], respectively.

3.4 Intervention characteristics

Table 3 presents the intervention characteristics of the thirteen included studies, focusing on three aspects: intervention type, frequency, and length. Intervention type: All studies primarily utilized HIFT as the primary intervention method. One study exclusively employed HIFT [61], while ten studies employed CrossFit [41,59,60,62,63,65–67,69,70]. One study combined CrossFit training with other methods like Rope-Skipping and Ki-Bo [64], and another study conducted comparative experiments between classic CrossFit and CrossFit AMRAP [68]. Intervention frequency: Most studies reported intervention frequency, with only one study omitting this information [69]. Among the remaining twelve studies, the intervention frequency ranged from two to four times per week [41,59–67,69,70]. Intervention length: All studies reported the duration of the intervention. The majority of experiments lasted between 4 and 12 weeks [41,59,61,63,65,68–70]. However, one study had a duration of five months [60], another study lasted for six months [66], one study lasted for nine months [64], and one study lasted for ten months [67].

3.5 Outcome

3.5.1 Effect of high-intensity functional training on strength of athletes. Six out of 13 studies examined the effects of HIFT on muscle strength in athletes [59,60,64,65,69,70]. These

Table 3. Summary of the studies' characteristics included in this review.

Study	Participant	Sex	Age	Exercise level	Intervention characteristics			Outcomes
					Type	Frequency	Length	
Mischenko et al. (2021) [64]	20	F	16–17 years	Professional WTF taekwondo athletes	EG: CrossFit, Rope-skipping, Tai-bo, Ki-bo, and Fightball Training CG: Routine Training	3 times/ week	9 months	SS↑ Running 100 m↑ PU↑ 1 min RS↑ SLJ↑ Attack speed↑
Osipov et al. (2019) [67]	31	M	16–17 years	Elite judo athletes	EG: CrossFit Training CG: Circular Training	3 times/ week	10 months	Competition score↑
Yüksel et al. (2019) [70]	32	M	21.72 ±1.40 years	Amateur wrestlers	EG: CrossFit Training CG: Classical Wrestling Training	3 times/ week,	8 weeks	Bench press↑ Squat jump↑
Ambroży et al. (2022) [59]	60	M	20.07 ± 1.46 years	Professional kickboxers	EG: CrossFit Training CG: Conventional Kickboxing Training	3 times/ week	8 weeks	Dynamic sit-ups↑ SLJ↑ Tapping↑ Cooper test↑ SR↑ Shuttle run 10x5 m↑ Movement speed↑
Kudryavtsev et al. (2023) [63]	44	M	21.06±3.42 years	Elite sambo athletes	EG: CrossFit Training CG: Strength Training	3 times/ week	4 weeks	Rank position↑
Zhu. (2023) [69]	24	NR	19.824 ±0.469 years	Professional aerobic gymnastics athletes	EG: CrossFit training CG: Normal aerobics training	NR	9 weeks	STMB↑ SR↑ Movement performance score↑
Osipov et al. (2022) [65]	53	M	17.22±1.37 years	Elite judo athletes	EG: CrossFit Training CG: Strength Training	4 times/ week	8 weeks	Pull up↑
Galimova et al. (2018) [62]	40	NR	18–19 years	College boxer	EG: CrossFit Training CG: Traditional training	4 times/ week	12 weeks	Attack strength↑
Türker & Yüksel. (2020) [68]	32	M	20.8 ± 1.15 years	Professional wrestlers	EG1: Classic CrossFit EG2: CrossFit AMRAP	3 times/ week	8 weeks	Anaerobic power↑
Caloglu & Yuksel. (2020) [41]	40	M	21.33 ± 1.78 years	Professional wrestlers	EG: CrossFit Training CG: No additional training	3 times/ week	8weeks	Anaerobic power↑ Dynamic balance↑
Avetisyan et al. (2022) [60]	20	M	11 ± 0.64 years	Junior judo athletes	EG: CrossFit Training CG: Traditional Training	2 times/ week	5 months	Pull-up↑ SLJ↑ Shuttle run time 4 × 10 m↔ Burpees (repetitions in 30 s) ↔

(*Continued*)

Table 3. (Continued)

Study	Participant	Sex	Age	Exercise level	Intervention characteristics			Outcomes
					Type	Frequency	Length	
Bozdoğan. (2021) [61]	22	F	16.41±1.29 years	Professional Volleyball Players	EG: HIFT CG: Routine training	2 times/ week	12 weeks	Shuttle run10 x 20 m↑ CMJ↑
Osipov et al. (2017) [66]	60	NR	20–21 years	Professional martial arts athletes	EG: CrossFit Training CG: Circular and Interval Training	2 times/ week	6 months	Competition endurance↑

M, male; F, female; NR, no record; EG, experiment group; CG, control group; HIFT = high-intensity functional training; SS, side split; PU, pull up; RS, rope skipping; SLJ, standing long jump; SR, sit and reach; STMB, side throw medicine ball; CMJ, counter movement jump. ↑, significant improvement; ↔, no significant difference.

https://doi.org/10.1371/journal.pone.0295531.t003

studies used various methods to evaluate strength indicators, including squats, bench presses, pull-ups, side throw medicine balls, and leg lifts. The results of this meta-analysis indicate that HIFT has a small effect on upper body muscle strength, in athletes (ES = 0.414; 95% CI = 0.14–0.683; p = 0.003; Egger test p = 0.170; N = 209; Fig 2A). There was low heterogeneity in the overall effect (Q = 5.044; I^2 = 0.872%). The relative weight of each study ranged from 9.69% to 29.03% during the analysis process. Furthermore, HIFT also significantly impacted lower body muscle strength, in athletes (ES = 1.051; 95% CI = 0.126–1.977; p = 0.026; Egger test p = 0.160; N = 177; Fig 2B). There was low heterogeneity in the overall effect (Q = 3.781; I^2 = 0.00%). Each study's relative weight ranged from 18.37% to 21.91% during the analysis.

3.5.2 Effect of high-intensity functional training on power of athletes. Seven studies investigated the impact of HIFT on power in a total of 184 male martial arts athletes, 20 female martial arts athletes, and 22 female volleyball athletes [41,59–61,64,68,70]. The main methods used to evaluate power included the squatting jump, standing long jump, and Anaerobic Power Test. The results of this meta-analysis indicate that HIFT has a small effect on power, among athletes (ES = 0.499; 95% CI = 0.239–0.758; p<0.001; Egger test p = 0.084; n = 226; Fig 3). In addition, low heterogeneity in the overall effect (Q = 4.676; I^2 = 0.00%) was also reported. Each study's relative weight ranged from 9.37% to 26.83% during the analysis.

3.5.3 Effect of high-intensity functional training on endurance of athletes. Four studies investigated the impact of HIFT on the endurance of 22 female volleyball athletes and 100 female martial arts athletes [59–61,64]. The results of this meta-analysis indicate that HIFT has a moderate but no significantly effect on endurance (ES = 0.798; 95% CI = -0.352–1.949; p = 0.174; Egger test p = 0.274; N = 122; Fig 4). There was low heterogeneity in the overall effect (Q = 3.765; I^2 = 20.245%). Each study's relative weight ranged from 22.67% to 27.57% during the analysis.

3.5.4 Effect of high-intensity functional training on agility of athletes. Three studies investigated the impact of HIFT on the agility of 80 male and 20 female martial arts athletes [59,60,64]. The results of this meta-analysis indicate that HIFT has no significant effect on athletes' agility performance (ES = 0.247; 95% CI = -0.443–0.537; p = 0.482; Egger test p = 0.601; N = 100; Fig 5). There was low heterogeneity in the overall effect (Q = 2.307; I^2 = 13.315%). Each study's relative weight ranged from 27.96% to 42.08% during the analysis.

3.5.5 Effect of high-intensity functional training on flexibility of athletes. Three studies investigated the impact of HIFT on the flexibility of 24 aerobic gymnasts and 80 martial arts athletes [59,64,69]. The results of this meta-analysis indicate that HIFT has a large

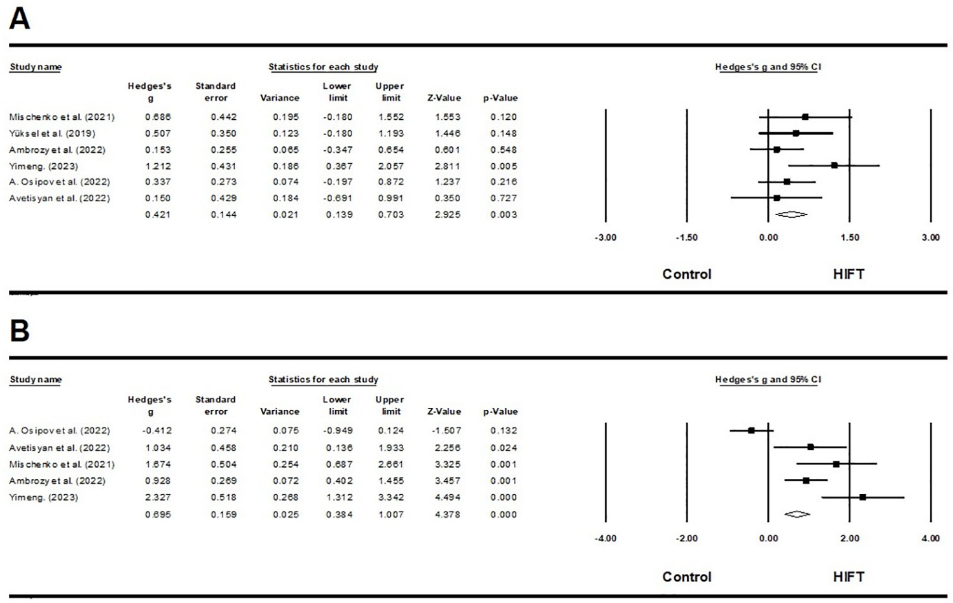

Fig 2. A. Forest plot of the effects of HIFT and control group on upper body strength. The displayed result is an effect size of 95% Cls. HIFT = high-intensity functional training. B. Forest plot of the effects of HIFT and control group on lower body strength. The displayed result is an effect size of 95% Cls. HIFT = high-intensity functional training.

https://doi.org/10.1371/journal.pone.0295531.g002

effect on athletes' flexibility performance (ES = 3.167; 95% CI = 0.477–5.857; p = 0.021; Egger test p = 0.029; N = 104; Fig 6). High heterogeneity exists in the overall effect (Q = 15.409; I^2 = 87.021%). Each study's relative weight ranged from 19.39% to 40.79% during the analysis.

3.5.6 Effect of high-intensity functional training on sport-specific performance of athletes. Seven studies investigated the impact of HIFT on the sport performance of 24 aerobic gymnasts and 255 martial arts athletes [59,62–64,66,67,69]. The results of this meta-analysis indicate that HIFT has a large effect on athletes' sport-specific performance (ES = 3.351; 95% CI = 1.780–4.922; p = 0.000; Egger's test p = 0.005; N = 279; Fig 7). There is no significant heterogeneity in the overall effect (Q = 6.784; I^2 = 11.554%). Each study's relative weight ranged from 12.73% to 15.25% during the analysis.

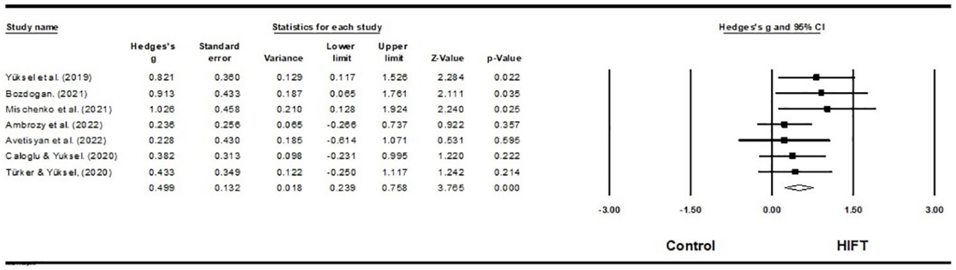

Fig 3. Forest plot of the effects of HIFT and control group on power. The displayed result is an effect size of 95% Cls. HIFT = high-intensity functional training.

https://doi.org/10.1371/journal.pone.0295531.g003

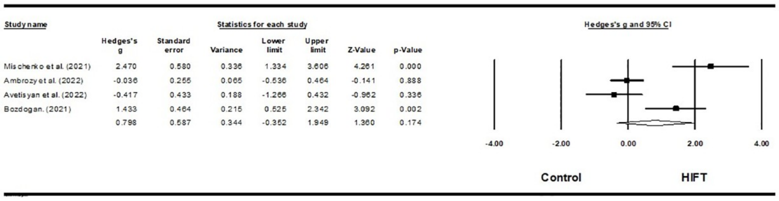

Fig 4. Forest plot of the effects of HIFT and control group on endurance. The displayed result is an effect size of 95% Cls. HIFT = high-intensity functional training.

https://doi.org/10.1371/journal.pone.0295531.g004

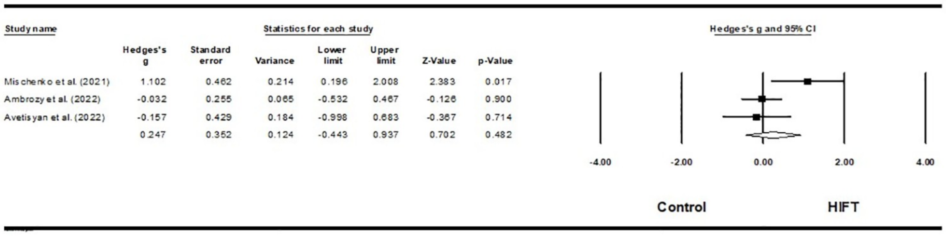

Fig 5. Forest plot of the effects of HIFT and control group on agility. The displayed result is an effect size of 95% Cls. HIFT = high-intensity functional training.

https://doi.org/10.1371/journal.pone.0295531.g005

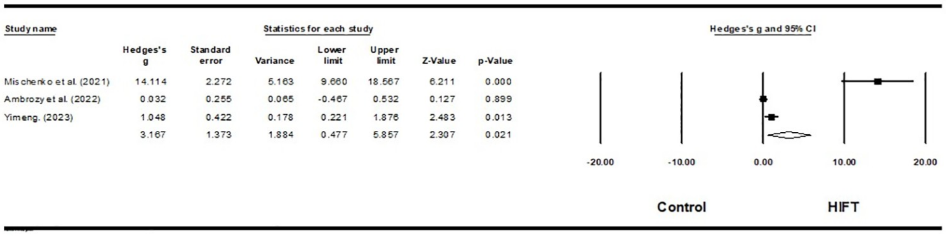

Fig 6. Forest plot of the effects of HIFT and control group on flexibility. The displayed result is an effect size of 95% Cls. HIFT = high-intensity functional training.

https://doi.org/10.1371/journal.pone.0295531.g006

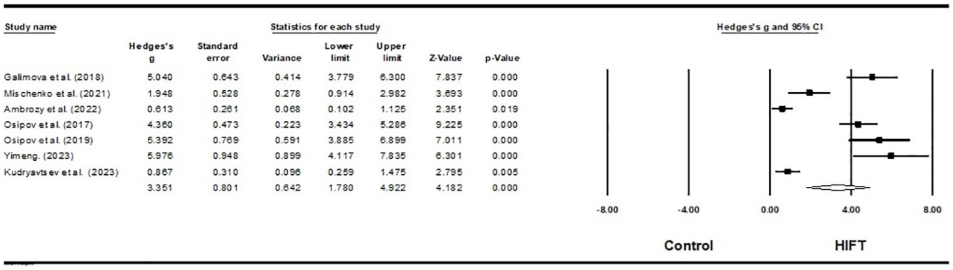

Fig 7. Forest plot of the effects of HIFT and control group on sport-specific performance. The displayed result is an effect size of 95% Cls. HIFT = high-intensity functional training.

https://doi.org/10.1371/journal.pone.0295531.g007

4 Discussion

4.1 Effect of high-intensity functional training on muscle strength of athletes

Numerous studies have indicated that combining resistance training and endurance training may lead to interference effects and impact athletes' training outcomes [72–76]. However, when athletes engage in HIFT training, this interference effect may not be observed [77]. A meta-analysis was conducted to assess the effects of HIFT on both upper-body muscle strength (ES = 0.414) and lower-body muscle strength (ES = 1.051). In this systematic review, six studies evaluated athletes' upper-body muscle strength in taekwondo, wrestling, judoka, and aerobic gymnastics. These findings are consistent with De Sousa et al., who investigated the effects of HIFT on lower limb muscle strength in young men [78]. Barfield et al. 2012 suggested that HIFT training is more effective in enhancing participants' lower limb muscle strength than upper limb strength. In contrast to previous reports, Heinrich et al. found through 1RM testing that the experimental group showed more significant improvement in upper limb muscle strength after HIFT intervention [10]. During muscle contraction, c-Jun N-terminal kinase (JNK) is activated. HIFT training stimulates an increase in JNK activity, promoting protein synthesis, which may increase muscle strength [79]. Previous research has demonstrated that the weightlifting component in HIFT training plans can significantly increase participants' muscle strength [10,78,80]. During the HIFT training process, participants often reach the point of muscle failure, increasing muscle strength at various repetition levels. Yüksel et al. found that eight weeks of HIFT improved the push-up performance of amateur wrestlers [70].

Muscle strength plays a crucial role in various movements performed by taekwondo athletes, such as horizontal kicks, whirlwind kicks, and rapid movements [81,82]. Mischenko et al. reported that female taekwondo athletes significantly improved in modified pull-ups after nine months of HIFT intervention [64]. Interestingly, one study included in this review compared the effects of high-intensity CrossFit training with traditional taekwondo training and found that the CrossFit training group significantly impacted athletes' pull-ups and push-ups. However, there was no significant difference in pull-ups compared to the control group, and push-ups showed significant statistical differences [59]. A recent study demonstrated that nine weeks of CrossFit training improved aerobic gymnasts' side-throw medicine ball performance [69]. Osipov et al. reported that eight weeks of HIFT significantly improved muscle strength performance in elite judo athletes, but no significant statistical difference was observed in lower limb strength compared to the control group in the squat test [65]. Therefore, the results of our meta-analysis indicate that HIFT is an effective method for enhancing athletes' upper and lower limb muscle strength performance.

4.2 Effect of high-intensity functional training on power of athletes

Numerous studies suggest that HIFT training may directly lead to improvements in power by enhancing muscle strength [10,78,83]. The meta-analysis revealed that HIFT had a significantly moderate impact on athletes' strength performance compared to the control group (ES = 0.499). Compared to traditional strength training, these improvements in power measurement align more closely with the principle of training specificity [84–86]. The effectiveness of power training for athletes can be enhanced by modifying the prescription of HIFT training [32,46]. Power refers to the ability of the human body to generate maximum force explosively following rapid muscle contractions, and it can increase with the duration of force development [87]. According to the force-velocity curve, different training methods within various ranges can enhance the rate of force development at each point on the curve, thereby

maximizing the benefits of power development [88,89]. Previous meta-analyses have also indicated that strength training improves adolescent athletic performance [90]. Within this systematic review, six studies evaluated the impact of HIFT on the strength performance of male Wushu athletes and female volleyball athletes, and significant improvements were observed. Current research suggests that HIFT can effectively enhance athletes' strength. These findings are consistent with the findings of Adami et al. The latter compared the physiological characteristics of HIFT, endurance, and power athletes and discovered the role of HIFT in enhancing male power [91]. Adami et al. examined the physiological characteristics of 20 HIFT athletes and found that they performed remarkably well in power-related aspects [92]. Notably, Caloglu and Yuksel found that eight weeks of HIFT positively impacted the power indicators of wrestlers [41]. Furthermore, numerous studies across different sports have demonstrated the effectiveness of HIFT in enhancing athletes' power ability [60,61,64,68,70]. Thus, our meta-analysis provides robust evidence supporting the positive impact of HIFT on athletes' power ability.

4.3 Effect of high-intensity functional training on endurance of athletes

Endurance is crucial for athletes to maintain high-level sports performance over an extended period [93]. The meta-analysis indicates that HIFT has a medium impact on the endurance performance of athletes (ES = 0.831). For sports with longer competitive durations, endurance directly determines athletes' outcomes in competitions, especially in sports like soccer [94], swimming [95], cycling [96], basketball [97], boxing [98], and judo [99]. HIFT is an effective alternative to traditional endurance training methods [91,100]. Training at high intensities with various movement patterns effectively promotes musculoskeletal, metabolic adaptations [101]. The human body adapts by increasing muscle fiber size and quantity to meet the demands of high-intensity loads, as well as enhancing the cardiovascular system's oxygen delivery capacity, thereby improving athletes' endurance levels for sustained exercise [102–104]. Four studies included in this systematic review examined the effects of HIFT on the endurance performance of martial arts athletes. This positive impact can be attributed to enhancing muscle endurance, lactate tolerance, and cardiorespiratory function in athletes following high-intensity functional training. Specifically, the short and intense aerobic interval training common in HIFT and the multi-mode high-intensity repetitive training help improve the cardiovascular system's adaptability, effectively delivering oxygen and nutrients to muscle tissues while also delaying lactate accumulation and enhancing athletes' lactate tolerance. Previous research has shown that cardiorespiratory function, exercise economy, and lactate may influence athletes' performance [105]. Additionally, Armstrong and Barker found that elite young athletes exhibit more substantial cardiorespiratory function [102]. Adami et al. found that HIFT effectively enhances athletes' endurance capacity [91]. Green et al. conducted a cross-sectional assessment of physiological differences among HIFT, endurance, and strength participants and found similar endurance performance between HIFT participants and endurance-trained athletes [106]. However, Gavanda et al. discovered that compared to traditional strength and endurance training, HIFT can effectively improve endurance capacity in adolescents [107]. This result aligns with the findings of Bozdoğan's study, which reported the positive effects of twelve weeks of high-intensity training on the aerobic endurance of volleyball athletes [61]. Ambroży et al. assessed the Cooper test results of 60 male taekwondo athletes and observed significant improvements in the 12-minute non-stop running performance following the intervention of HIFT [59].

4.4 Effect of high-intensity functional training on agility of athletes

Agility is the ability of athletes to react quickly in a short period [108]. Our meta-analysis indicates that the impact of HIFT on athletes' agility performance can be considered negligible (ES = 0.247). HIFT typically involves various fast and explosive movements, especially clean and jerk, and box jump training [26]. These movements require rapid force generation after cutting off the force, enhancing the neural capacity for quick reactions, and enabling the body to respond accurately in fast-paced environments [109]. Gary Grey's "Chain Reaction Exploratory Theory" is strongly linked to agility training for athletes [110]. Agility is typically demonstrated by an athlete's ability to control body posture and rapidly change directions [111]. Crucial factors affecting agility include speed, muscle strength [112,113], and balance [114,115]. Possessing good agility helps athletes perform various actions more quickly in competitions, better control their body posture, and easily demonstrate personal skills, thereby increasing their competitive advantage [116]. HIFT emphasizes multi-joint and high-intensity movements, which positively affect the human body's neural-muscular coordination and proprioceptive abilities [31]. Three studies included in this systematic review evaluated the agility performance of volleyball and martial arts athletes. These findings are consistent with the results reported by Carvutto et al. Carvutto et al. reported significant improvements in athletes' agility performance, although there was no statistically significant difference compared to the control group [71]. Additionally, Hovsepian et al. found significant improvements in agility performance for both the experimental and control groups of athletes after HIFT training, although there were no statistically significant differences between the two groups [14]. However, Ambroży et al. discovered that after eight weeks of HIFT, the shuttle run performance of taekwondo athletes had improved, and the study also highlighted the significant correlation between the enhancement of shuttle run performance and hip joint rotation speed as a specific quality [59]. Rapid movement is essential in combat and defence as the foundation for achieving technical and tactical goals [117].

4.5 Effect of high-intensity functional training on flexibility of athletes

Flexibility refers to the ability of muscles and joints to stretch, which is crucial for increasing the range of motion and muscle elasticity in the human body [118,119]. Our meta-analysis has demonstrated that HIFT significantly impacts athletes' flexibility performance (ES = 3.167), which refers to the specific range of muscle activity or muscle activity ability [119]. Through comprehensive multi-joint training, muscles and joints undergo adaptive changes due to stretching and compression, which enhances athletes' joint stability and muscle elasticity [120]. The sit-and-reach test is commonly used to measure flexibility [121]. Three studies included in this systematic review examined the impact of HIFT on the flexibility performance of aerobic gymnastics and martial arts athletes. These findings align with the results of Chizewski et al., who found that a seven-week HIFT training program effectively improved athletes' flexibility [38]. Good flexibility is beneficial for enhancing muscle efficiency [69]. Stretching exercises help increase muscle elasticity, activate the nervous system, and prevent sports injuries [122]. Furthermore, multiple studies emphasize the importance of flexibility for athletes' performance [122–127]. It is noteworthy that HIFT incorporates various functional movements, particularly clean and jerks, squats, and lunges, which may be crucial factors in improving athletes' joint flexibility [77]. Research conducted by Cosgrove et al. demonstrates that after six months of HIFT, both adult males and females showed improved flexibility [128]. Similarly, Zagdsuren et al. found that after ten weeks of HIFT, the flexibility of both men and women significantly increased [129].

4.6 Effect of high-intensity functional training on sport-specific performance of athletes

Sport-specific performance refers to athletes' proficiency and effectiveness in specific sports processes, forming the foundation for achieving outstanding competition results [130]. Our meta-analysis has revealed that HIFT significantly impacts athletes' sport-specific performance (ES = 3.351). The mastery of sport-specific techniques and the skillful application of tactics enable athletes to better leverage their technical advantages and make informed decisions [131]. For example, fast attacking speed allows athletes to seize opportunities when opponents' defences still need to be established [132]. Strong attacking power enables athletes to execute more powerful offensive actions, exerting pressure on opponents [133]. This systematic review includes seven studies examining the impact of HIFT on athletes' technical performance. De Sousa et al.'s research demonstrated that HIFT significantly improved vertical jump performance in male individuals [78]. Hermassi et al. indicated that HIFT programs effectively enhanced the vertical jump performance of high-level handball athletes [83]. Similarly, Hilaiel and Alsulan found that HIFT plans effectively improved the shooting accuracy of five-a-side soccer players [39]. Previous research has shown that HIFT enhances athletes' speed and attack accuracy [134]. The development of overall physical fitness is crucial for athletes to utilize techniques effectively and enhance their competitive performance [135,136]. Mischenko et al. discovered that young women WTF taekwondo athletes significantly increased their attack speed and combat endurance after nine months of HIFT [64]. Özgür et al. reported that taekwondo athletes' vertical jump performance significantly improved after HIFT training [137]. Galimova et al. reported that a twelve-week HIFT program significantly improved the attacking power of university boxers [62]. Additionally, Pawlak et al. (2015) found that a 12-week HIFT intervention significantly enhanced firefighters' performance in simulated firefighting tasks.

5 Limitations

This meta-analysis has several limitations that should be acknowledged:

1. The minimal number of studies (n = 13) included in this review may restrict the generalizability of the findings. Ideally, a meta-analysis should include at least three studies for each analysis, but some of the analyses in this review were based on fewer studies.
2. Only one study met the inclusion criteria for screening literature. Therefore, the analysis of this outcome was limited.
3. The training protocols used in the included studies varied, with different types of HIFT, such as CrossFit, Fan, and Cindy, which may lead to some degree of inconsistency in the results.
4. Some studies needed to provide detailed information on the training protocols, including the duration of each training session, which could have influenced the results.
5. This meta-analysis focused primarily on overall physical abilities and did not specifically examine technical performance or combat ability, which are essential aspects of athletes' overall performance.

6 Conclusions

In conclusion, this systematic review and meta-analysis suggest that HIFT positively impacts athletes' physical abilities and specific performance in sports such as aerobic gymnastics,

volleyball, taekwondo, judoka, sambo, and wrestling. HIFT effectively improves athletes' muscle strength, power, flexibility, and sport-specific performance but has no significant impact on endurance and agility. HIFT can stimulate neuroendocrine responses [43], triggering increased recruitment of muscles [30,44], consequently enhancing athletic performance. Coaches, trainers, and athletes can refer to the results of this meta-analysis to guide training programs and optimize performance results. However, due to the limited number of studies and the heterogeneity in training protocols, further research is needed to explore the potential variables that affect athletes' physical fitness and specialized performance in the HIFT intervention process. Additionally, future studies should investigate the effects of the HIFT intervention program on athletes' speed, balance, and technical and tactical performance parameters to understand better its impact on overall physical fitness and specialized abilities.

7 Practical application

The results of this systematic review and meta-analysis have significant implications for athletes, coaches, and sports professionals seeking to improve athletes' physical fitness and specific sports performance. Additionally, this training program requires a relatively short time commitment and recruits more muscle groups than single-mode HIIT training [31]. The diversity and dynamism of HIFT training contribute to athletes' rapid adaptation to specialized movements. The current systematic review includes limited studies, and no subgroup analysis of gender, age, or training variables was conducted. This review suggests that coaches and trainers incorporate this mode into athletes' training plans, with a frequency of at least two sessions per week and a duration of at least four weeks. Additionally, considering additional training strategies targeting specific aspects such as endurance, agility, speed, and balance, as well as technical and tactical performance, are recommended.

Supporting information

S1 Checklist. PRISMA 2020 checklist.
(DOCX)

Author Contributions

Conceptualization: Xinzhi Wang, Kim Geok Soh, Shamsulariffin Samsudin, Nuannuan Deng.

Data curation: Xinzhi Wang.

Formal analysis: Xinzhi Wang, Nuannuan Deng.

Investigation: Xinzhi Wang, Shamsulariffin Samsudin.

Methodology: Xinzhi Wang, Kim Geok Soh, Nuannuan Deng, Yue Zhao.

Resources: Xinzhi Wang.

Software: Xinzhi Wang.

Supervision: Kim Geok Soh, Shamsulariffin Samsudin, Nuannuan Deng, Xutao Liu, Yue Zhao.

Validation: Xinzhi Wang, Kim Geok Soh, Nuannuan Deng, Xutao Liu, Yue Zhao, Saddam Akbar.

Writing – original draft: Xinzhi Wang.

Writing – review & editing: Xinzhi Wang, Kim Geok Soh, Shamsulariffin Samsudin, Nuannuan Deng, Xutao Liu, Yue Zhao, Saddam Akbar.

References

1. Haddock CK, Poston WSC, Heinrich KM, Jahnke SA, Jitnarin N. The Benefits of High-Intensity Functional Training Fitness Programs for Military Personnel. Mil Med. 2016 Nov; 181(11):e1508–14. https://doi.org/10.7205/MILMED-D-15-00503 PMID: 27849484
2. Butcher SJ, Neyedly TJ, Horvey KJ, Benko CR. Do physiological measures predict selected CrossFit® benchmark performance? Open access journal of sports medicine. 2015;241–7.
3. Chukhlantseva N, Cherednychenko I, Kemkina V. The influence of high-intensity functional training versus resistance training on the main physical fitness indicators in women aged 25–35 years. Trends in Sport Sciences [Internet]. 2020 Jul [cited 2023 Feb 22]; 27(3):157–65. Available from: https://search.ebscohost.com/login.aspx?direct=true&db=s3h&AN=146234539&site=ehost-live.
4. Claudino JG, Gabbett TJ, Bourgeois F, Souza H de S, Miranda RC, Mezêncio B, et al. CrossFit Overview: Systematic Review and Meta-analysis. Sports Med Open. 2018 Feb 26; 4(1):11. https://doi.org/10.1186/s40798-018-0124-5 PMID: 29484512
5. Poston WS, Haddock CK, Heinrich KM, Jahnke SA, Jitnarin N, Batchelor DB. Is high-intensity functional training (HIFT)/CrossFit safe for military fitness training? Military medicine. 2016; 181(7):627–37. https://doi.org/10.7205/MILMED-D-15-00273 PMID: 27391615
6. Smith MM, Sommer AJ, Starkoff BE, Devor ST. Crossfit-Based High-Intensity Power Training Improves Maximal Aerobic Fitness and Body Composition [RETRACTED]. The Journal of Strength & Conditioning Research. 2013; 27(11):3159–72.
7. Thompson WR. Worldwide survey of fitness trends for 2020. ACSM's Health & Fitness Journal. 2019; 23(6):10–8.
8. Feito Y, Hoffstetter W, Serafini P, Mangine G. Changes in body composition, bone metabolism, strength, and skill-specific performance resulting from 16-weeks of HIFT. PLoS One. 2018; 13(6): e0198324. https://doi.org/10.1371/journal.pone.0198324 PMID: 29906290
9. Feito Y, Moriarty TA, Mangine G, Monahan J. The use of a smart-textile garment during high-intensity functional training: a pilot study. J Sports Med Phys Fitness. 2019 Jun; 59(6):947–54. https://doi.org/10.23736/S0022-4707.18.08689-9 PMID: 30024125
10. Heinrich KM, Spencer V, Fehl N, Carlos Poston WS. Mission essential fitness: comparison of functional circuit training to traditional Army physical training for active duty military. Military medicine. 2012; 177(10):1125–30. https://doi.org/10.7205/milmed-d-12-00143 PMID: 23113436
11. Kliszczewicz B, McKenzie M, Nickerson B. Physiological adaptation following four-weeks of high-intensity functional training. Vojnosanitetski pregled. 2019; 76(3):272–7.
12. Buckley S, Knapp K, Lackie A, Lewry C, Horvey K, Benko C, et al. Multimodal high-intensity interval training increases muscle function and metabolic performance in females. Applied Physiology, Nutrition, and Metabolism. 2015; 40(11):1157–62. https://doi.org/10.1139/apnm-2015-0238 PMID: 26513008
13. Franchini E, Brito CJ, Fukuda DH, Artioli GG. The physiology of judo-specific training modalities. The Journal of Strength & Conditioning Research. 2014; 28(5):1474–81. https://doi.org/10.1519/JSC.0000000000000281 PMID: 24149757
14. Hovsepian A, Esfarjani F, Bambaeichi E, Zolaktaf V. The effect of high intensity functional training on the oxidative status, muscle damage and performance of basketball players. The Journal of sports medicine and physical fitness. 2021; 61(2):188–98. https://doi.org/10.23736/S0022-4707.20.11094-6 PMID: 32674538
15. Bourgois JG, Dumortier J, Callewaert M, Celie B, Capelli C, Sjøgaard G, et al. Tribute to Dr Jacques Rogge: muscle activity and fatigue during hiking in Olympic dinghy sailing. European Journal of Sport Science. 2017; 17(5):611–20. https://doi.org/10.1080/17461391.2017.1300328 PMID: 28316262
16. Pan D, Zhong B, Guo W, Xu Y. Physical fitness characteristics and performance in single-handed dinghy and 470 classes sailors. Journal of Exercise Science & Fitness [Internet]. 2022 Jan 1 [cited 2023 Jul 20]; 20(1):9–15. Available from: https://www.sciencedirect.com/science/article/pii/S1728869X21000459 PMID: 34868325
17. Fernandez-Fernandez J, De Villarreal ES, Sanz-Rivas D, Moya M. The effects of 8-week plyometric training on physical performance in young tennis players. Pediatric exercise science. 2016; 28(1):77–86. https://doi.org/10.1123/pes.2015-0019 PMID: 26252503

18. Podrigalo L, Iermakov S, Romanenko V, Rovnaya O, Tropin Y, Goloha V, et al. Psychophysiological features of athletes practicing different styles of martial arts-the comparative analysis. International Journal of Applied Exercise Physiology. 2019; 8(1):84–91.

19. Sobrero G, Schafer M, Tolbert TA, Crandall J, Brown J, Esslinger T, et al. A Comparison of High Intensity Functional Training and Circuit Training on Health and Performance Variables in Women: A Pilot Study. Women in Sport & Physical Activity Journal [Internet]. 2017 Apr [cited 2023 Feb 22]; 25(1):1–10. Available from: https://search.ebscohost.com/login.aspx?direct=true&db=s3h&AN=123004325&site=ehost-live.

20. Young WB. Transfer of strength and power training to sports performance. International journal of sports physiology and performance. 2006; 1(2):74–83. https://doi.org/10.1123/ijspp.1.2.74 PMID: 19114741

21. D'Isanto T, D'Elia F, Raiola G, Altavilla G. Assessment of sport performance: Theoretical aspects and practical indications. Sport Mont. 2019; 17(1):79–82.

22. Fernandez-Fernandez J, García-Tormo V, Santos-Rosa FJ, Teixeira AS, Nakamura FY, Granacher U, et al. The effect of a neuromuscular vs. dynamic warm-up on physical performance in young tennis players. The Journal of Strength & Conditioning Research. 2020; 34(10):2776–84. https://doi.org/10.1519/JSC.0000000000003703 PMID: 32986392

23. Wang Y. Tracing the Theory of Human Motion Chain and Its Enlightenment for Functional Training. Journal of Chengdu Sport University. 2017; 43(2):60–6.

24. Islam S. Applied Fitness Training: A Practical Approach to Learning and Instructing Kinesiology [PhD Thesis]. California State University, Northridge; 2014.

25. Feito Y, Brown C, Olmos A. A content analysis of the High-Intensity Functional Training Literature: a look at the past and directions for the future. Human Movement. 2019; 20(2):1–15.

26. Box AG, Feito Y, Brown C, Heinrich KM, Petruzzello SJ. High Intensity Functional Training (HIFT) and competitions: How motives differ by length of participation. PLoS One. 2019; 14(3):e0213812. https://doi.org/10.1371/journal.pone.0213812 PMID: 30897101

27. Heinrich KM, Becker C, Carlisle T, Gilmore K, Hauser J, Frye J, et al. High-intensity functional training improves functional movement and body composition among cancer survivors: a pilot study. European journal of cancer care. 2015; 24(6):812–7. https://doi.org/10.1111/ecc.12338 PMID: 26094701

28. Murawska-Cialowicz E, Wojna J, Zuwala-Jagiello J. Crossfit training changes brain-derived neurotrophic factor and irisin levels at rest, after wingate and progressive tests, and improves aerobic capacity and body composition of young physically active men and women. J Physiol Pharmacol. 2015; 66(6):811–21. PMID: 26769830

29. Senefeld JW, Joyner MJ. Strength-Endurance Training Classes: Health Benefits and Injury Rates of an Emerging Cornerstone of Physical Activity. Mayo Clinic Proceedings [Internet]. 2020 Mar 1 [cited 2023 Apr 26]; 95(3):437–9. Available from: https://www.sciencedirect.com/science/article/pii/S0025619620300707 PMID: 32138874

30. Wilke J, Mohr L. Chronic effects of high-intensity functional training on motor function: a systematic review with multilevel meta-analysis. Sci Rep. 2020 Dec 10; 10(1):21680. https://doi.org/10.1038/s41598-020-78615-5 PMID: 33303848

31. Feito Y, Heinrich KM, Butcher SJ, Poston WSC. High-Intensity Functional Training (HIFT): Definition and Research Implications for Improved Fitness. Sports (Basel). 2018 Aug 7; 6(3):76. https://doi.org/10.3390/sports6030076 PMID: 30087252

32. Crawford DA, Drake NB, Carper MJ, DeBlauw J, Heinrich KM. Are changes in physical work capacity induced by high-intensity functional training related to changes in associated physiologic measures? Sports. 2018; 6(2):26. https://doi.org/10.3390/sports6020026 PMID: 29910330

33. Dexheimer JD, Brinson SJ, Pettitt RW, Schroeder ET, Sawyer BJ, Jo E. Predicting Maximal Oxygen Uptake Using the 3-Minute All-Out Test in High-Intensity Functional Training Athletes. Sports (2075–4663) [Internet]. 2020 Dec [cited 2023 Feb 22]; 8(12):155. Available from: https://search.ebscohost.com/login.aspx?direct=true&db=s3h&AN=147795243&site=ehost-live PMID: 33266118

34. Tibana RA, De Sousa NMF, Cunha GV, Prestes J, Fett C, Gabbett TJ, et al. Validity of session rating perceived exertion method for quantifying internal training load during high-intensity functional training. Sports. 2018; 6(3):68. https://doi.org/10.3390/sports6030068 PMID: 30041435

35. Meyer J, Morrison J, Zuniga J. The benefits and risks of CrossFit: a systematic review. Workplace health & safety. 2017; 65(12):612–8. https://doi.org/10.1177/2165079916685568 PMID: 28363035

36. Montalvo AM, Shaefer H, Rodriguez B, Li T, Epnere K, Myer GD. Retrospective Injury Epidemiology and Risk Factors for Injury in CrossFit. J Sports Sci Med. 2017 Mar; 16(1):53–9. PMID: 28344451

37. Neto JHF, Kennedy MD. The Multimodal Nature of High-Intensity Functional Training: Potential Applications to Improve Sport Performance. Sports (Basel). 2019 Jan 29; 7(2):33. https://doi.org/10.3390/sports7020033 PMID: 30699906

38. Chizewski A, Box A, Kesler RM, Petruzzello SJ. High Intensity Functional Training (HIFT) Improves Fitness in Recruit Firefighters. International Journal of Environmental Research and Public Health [Internet]. 2021 Jan [cited 2022 Dec 8]; 18(24):13400. Available from: https://www.mdpi.com/1660-4601/18/24/13400 PMID: 34949008

39. Hilaiel DSC, Alsulan MSA. The Effect of a Training Curriculum Using High-Intensity Functional Training (HIFT) on Some Physical Abilities and Scoring Accuracy for Futsal Football Players. Annals of the Romanian Society for Cell Biology [Internet]. 2021 Aug 20 [cited 2023 Jul 31]; 25(6):19652–61. Available from: http://annalsofrscb.ro/index.php/journal/article/view/9820.

40. Wibowo S, Nurhasan, Fathir LW, Ashadi K, Hartoto S, Ardha MAA, et al. The effect of a short term high intensity functional strength training on strength and endurance in recreational runners. Journal of Physical Education & Sport [Internet]. 2021 Aug 2 [cited 2023 Feb 22]; 21:2332–6. Available from: https://search.ebscohost.com/login.aspx?direct=true&db=s3h&AN=152939770&site=ehost-live.

41. Caloglu M, Yuksel O. The Effect of Cross Fit Training on Anaerobic Power and Dynamic Balance of Greco-Roman and Freestyle Wrestlers. Int J Appl Exerc Physiol [Internet]. 2020 Jan [cited 2023 May 12]; 9(1):122–32. Available from: https://www.webofscience.com/wos/alldb/summary/9c9977bd-6b17-498b-a6f6-c5ee11b1a3b6-8964feab/relevance/1.

42. Ben-Zeev T, Hirsh T, Weiss I, Gornstein M, Okun E. The Effects of High-intensity Functional Training (HIFT) on Spatial Learning, Visual Pattern Separation and Attention Span in Adolescents. Front Behav Neurosci. 2020; 14:577390. https://doi.org/10.3389/fnbeh.2020.577390 PMID: 33093827

43. Glassman G. A Beginner's Guide to CrossFit. CrossFit Journal. 2004; 26:1–5.

44. Heinrich KM, Crawford DA, Langford CR, Kehler A, Andrews V. High-Intensity Functional Training Shows Promise for Improving Physical Functioning and Activity in Community-Dwelling Older Adults: A Pilot Study. Journal of Geriatric Physical Therapy. 2021; 44(1):9–17. https://doi.org/10.1519/JPT.0000000000000251 PMID: 31626033

45. Bustos-Viviescas BJ, Ramírez-Campillo R, Aguirre-Rueda DM, Merchán Osorio RD, García Yerena CE, Acevedo Mindiola AA. High-intensity functional training and quantification by Perceived Exertion Scale in physically active subjects. / Entrenamiento funcional de alta intensidad y su cuantificación por Escala de Esfuerzo Percibido en sujetos físicamente activos. Cultura, Ciencia y Deporte [Internet]. 2022 Jan [cited 2022 Dec 10]; 17(51):153–67. Available from: https://search.ebscohost.com/login.aspx?direct=true&db=s3h&AN=155543277&site=ehost-live.

46. Kliszczewicz B, Williamson C, Bechke E, McKenzie M, Hoffstetter W. Autonomic response to a short and long bout of high-intensity functional training. Journal of Sports Sciences. 2018; 36(16):1872–9. https://doi.org/10.1080/02640414.2018.1423857 PMID: 29308709

47. Brisebois MF, Rigby BR, Nichols DL. Physiological and Fitness Adaptations after Eight Weeks of High-Intensity Functional Training in Physically Inactive Adults. Sports (Basel). 2018 Nov 13; 6(4):146. https://doi.org/10.3390/sports6040146 PMID: 30428527

48. McKenzie JE, Brennan SE, Ryan RE, Thomson HJ, Johnston RV, Thomas J. Defining the criteria for including studies and how they will be grouped for the synthesis. In: Cochrane Handbook for Systematic Reviews of Interventions [Internet]. John Wiley & Sons, Ltd; 2019 [cited 2023 Aug 9]. p. 33–65. https://onlinelibrary.wiley.com/doi/abs/10.1002/9781119536604.ch3.

49. Egger M, Juni P, Bartlett C, Holenstein F, Sterne J. How important are comprehensive literature searches and the assessment of trial quality in systematic reviews? Empirical study. Health technol assess. 2003; 7(1):1–76. PMID: 12583822

50. Higgins JP, Thomas J, Chandler J, Cumpston M, Li T, Page MJ, et al. Cochrane handbook for systematic reviews of interventions. John Wiley & Sons; 2019.

51. Hozo SP, Djulbegovic B, Hozo I. Estimating the mean and variance from the median, range, and the size of a sample. BMC medical research methodology. 2005; 5(1):1–10. https://doi.org/10.1186/1471-2288-5-13 PMID: 15840177

52. Wan X, Wang W, Liu J, Tong T. Estimating the sample mean and standard deviation from the sample size, median, range and/or interquartile range. BMC medical research methodology. 2014; 14:1–13.

53. De Morton NA. The PEDro scale is a valid measure of the methodological quality of clinical trials: a demographic study. Australian Journal of Physiotherapy. 2009; 55(2):129–33. https://doi.org/10.1016/s0004-9514(09)70043-1 PMID: 19463084

54. Maher CG, Sherrington C, Herbert RD, Moseley AM, Elkins M. Reliability of the PEDro scale for rating quality of randomized controlled trials. Physical therapy. 2003; 83(8):713–21. PMID: 12882612

55. Borenstein M, Hedges LV, Higgins JP, Rothstein HR. A basic introduction to fixed-effect and random-effects models for meta-analysis. Research synthesis methods. 2010; 1(2):97–111. https://doi.org/10.1002/jrsm.12 PMID: 26061376

56. Hopkins W, Marshall S, Batterham A, Hanin J. Progressive statistics for studies in sports medicine and exercise science. Medicine+ Science in Sports+ Exercise. 2009; 41(1):3.

57. Higgins JP, Thompson SG, Deeks JJ, Altman DG. Measuring inconsistency in meta-analyses. Bmj. 2003; 327(7414):557–60. https://doi.org/10.1136/bmj.327.7414.557 PMID: 12958120

58. Egger M, Smith GD, Schneider M, Minder C. Bias in meta-analysis detected by a simple, graphical test. Bmj. 1997; 315(7109):629–34. https://doi.org/10.1136/bmj.315.7109.629 PMID: 9310563

59. Ambroży T, Rydzik Ł, Kwiatkowski A, Spieszny M, Ambroży D, Rejman A, et al. Effect of CrossFit Training on Physical Fitness of Kickboxers. Int J Environ Res Public Health. 2022 Apr 8; 19(8):4526. https://doi.org/10.3390/ijerph19084526 PMID: 35457394

60. Avetisyan AV, Chatinyan AA, Streetman AE, Heinrich KM. The Effectiveness of a CrossFit Training Program for Improving Physical Fitness of Young Judokas: A Pilot Study. Journal of Functional Morphology and Kinesiology. 2022; 7(4):83. https://doi.org/10.3390/jfmk7040083 PMID: 36278744

61. Bozdoğan T. The effects of high-intensity functional training on aerobic capacity, metabolic adaptation and neuromuscular responses in young female volleyball players. 2021 Dec 1 [cited 2023 Jun 7]; https://avesis.marmara.edu.tr/api/publication/a7c5f8a3-4a58-4d36-95eb-6ab8be360bbe/file.

62. Galimova A, Kudryavtsev M, Galimov G, Osipov A, Astaf'ev N, Zhavner T, et al. Increase in power striking characteristics via intensive functional training in crossfit. Journal of Physical Education & Sport [Internet]. 2018 Jun [cited 2023 Jan 6]; 18(2):585–91. Available from: https://search.ebscohost.com/login.aspx?direct=true&db=s3h&AN=130654866&site=ehost-live.

63. Kudryavtsev M, Osipov A, Guralev V, Ratmanskaya T, Aldiabat H, Aldiabat I, et al. Effect of short-term functional training intervention on athletic performance in elite male combat sambo athletes. Journal of Physical Education and Sport. 2023; 23(2):328–34.

64. Mischenko N, Kolokoltsev M, Vorozheikin A, Romanova E, Bolotin A, Aganov S, et al. An innovative package of training techniques effectiveness in Taekwondo. Journal of Physical Education and Sport. 2021; 21:3214–21.

65. Osipov A, Guralev V, Iermakov S, Ratmanskaya T, Galimova A, Kudryavtsev M. Impact of two different strength/conditioning training interventions on sport and strength performance of junior male judokas. Phys Act Rev [Internet]. 2022 [cited 2023 May 12]; 10(1):98–106. Available from: https://www.webofscience.com/wos/alldb/summary/9c9977bd-6b17-498b-a6f6-c5ee11b1a3b6-8964feab/relevance/1.

66. Osipov A, Kudryavtsev M, Gatilov K, Zhavner T, Klimuk Y, Ponomareva E, et al. The use of functional training–crossfit methods to improve the level of special training of athletes who specialize in combat sambo. Journal of Physical Education and Sport. 2017; 17(3):2013–8.

67. Osipov AY, Nagovitsyn RS, Zekrin FH, Vladimirovna FT, Zubkov DA, Zhavner TV. CrossFit Training Impact on the Level of Special Physical Fitness of Young Athletes Practicing Judo. Sport Mont [Internet]. 2019 Oct [cited 2023 Jan 6]; 17(3):9–12. Available from: https://search.ebscohost.com/login.aspx?direct=true&db=s3h&AN=139246450&site=ehost-live.

68. Türker A, Yüksel O. Investigation of the Effect of Amrap and Classic Crossfit Trainings in Wrestlers on Anerobic Power. International Journal of Applied Exercise Physiology. 2020; 9(9):73–81.

69. Zhu Y. Effects of crossfit training on body function and movement performance of aerobic athletes. Rev Bras Med Esporte [Internet]. 2023 Apr 7 [cited 2023 Jun 7]; 29:e2023_0019. Available from: https://www.scielo.br/j/rbme/a/GzwptvJQjCgz9mHKrDmPntN/.

70. Yüksel O, Gündüz B, Kayhan M. Effect of Crossfit Training on Jump and Strength. Journal of Education and Training Studies [Internet]. 2019 Jan [cited 2023 May 12]; 7(1):121–4. Available from: https://eric.ed.gov/?id=EJ1202023.

71. Carvutto R, Damasco C, De Candia M. Non-traditional training in youth soccer players: Effects on agility and on sprint performance. 2021 [cited 2023 Jun 7]; http://rua.ua.es/dspace/handle/10045/119242.

72. Coffey VG, Hawley JA. Concurrent exercise training: do opposites distract? The Journal of physiology. 2017; 595(9):2883–96. https://doi.org/10.1113/JP272270 PMID: 27506998

73. Fyfe JJ, Bishop DJ, Stepto NK. Interference between concurrent resistance and endurance exercise: molecular bases and the role of individual training variables. Sports medicine. 2014; 44:743–62. https://doi.org/10.1007/s40279-014-0162-1 PMID: 24728927

74. Murach KA, Bagley JR. Skeletal muscle hypertrophy with concurrent exercise training: contrary evidence for an interference effect. Sports medicine. 2016; 46:1029–39. https://doi.org/10.1007/s40279-016-0496-y PMID: 26932769

75. Wilson JM, Marin PJ, Rhea MR, Wilson SM, Loenneke JP, Anderson JC. Concurrent training: a meta-analysis examining interference of aerobic and resistance exercises. The Journal of Strength & Conditioning Research. 2012; 26(8):2293–307. https://doi.org/10.1519/JSC.0b013e31823a3e2d PMID: 22002517

76. Wong P lam, Chaouachi A, Chamari K, Dellal A, Wisloff U. Effect of preseason concurrent muscular strength and high-intensity interval training in professional soccer players. The Journal of Strength & Conditioning Research. 2010; 24(3):653–60. https://doi.org/10.1519/JSC.0b013e3181aa36a2 PMID: 19816215

77. Falk Neto JH, Kennedy MD. The multimodal nature of high-intensity functional training: potential applications to improve sport performance. Sports. 2019; 7(2):33. https://doi.org/10.3390/sports7020033 PMID: 30699906

78. de Sousa AF, dos Santos GB, dos Reis T, Valerino AJ, Del Rosso S, Boullosa DA. Differences in Physical Fitness between Recreational CrossFit® and Resistance Trained Individuals. Journal of Exercise Physiology Online. 2016; 19(5).

79. Ben-Zeev T, Okun E. High-Intensity Functional Training: Molecular Mechanisms and Benefits. Neuromolecular Med. 2021 Sep; 23(3):335–8. https://doi.org/10.1007/s12017-020-08638-8 PMID: 33386577

80. Barfield JP, Channell B, Pugh C, Tuck M, Pendel D. Format of basic instruction program resistance training classes: Effect on fitness change in college students. Physical educator. 2012; 69(4):325.

81. Bridge CA, Ferreira da Silva Santos J, Chaabene H, Pieter W, Franchini E. Physical and physiological profiles of taekwondo athletes. Sports Medicine. 2014; 44:713–33. https://doi.org/10.1007/s40279-014-0159-9 PMID: 24549477

82. Zabukovec R, Tiidus PM. Physiological and anthropometric profile of elite kickboxers. The Journal of Strength & Conditioning Research. 1995; 9(4):240–2.

83. Hermassi S, Wollny R, Schwesig R, Shephard RJ, Chelly MS. Effects of in-season circuit training on physical abilities in male handball players. The Journal of Strength & Conditioning Research. 2019; 33 (4):944–57. https://doi.org/10.1519/JSC.0000000000002270 PMID: 29016476

84. Behm DG. Neuromuscular implications and applications of resistance training. Journal of Strength and Conditioning Research. 1995; 9(4):264–74.

85. Behm DG, Sale DG. Velocity specificity of resistance training. Sports medicine. 1993; 15:374–88. https://doi.org/10.2165/00007256-199315060-00003 PMID: 8341872

86. Sale D, MacDougall D. Specificity in strength training: a review for the coach and athlete. Canadian journal of applied sport sciences Journal canadien des sciences appliquées au sport. 1981; 6(2):87–92. PMID: 7016357

87. Gerhart HD. A comparison of CrossFit training to traditional anaerobic resistance training in terms of selected fitness domains representative of overall athletic performance. Indiana University of Pennsylvania; 2013.

88. Haff GG, Nimphius S. Training principles for power. Strength & Conditioning Journal. 2012; 34(6):2–12.

89. Suchomel TJ, Comfort P, Lake JP. Enhancing the force-velocity profile of athletes using weightlifting derivatives. Strength & Conditioning Journal. 2017; 39(1):10–20.

90. Behm DG, Young JD, Whitten JH, Reid JC, Quigley PJ, Low J, et al. Effectiveness of traditional strength vs. power training on muscle strength, power and speed with youth: a systematic review and meta-analysis. Frontiers in physiology. 2017; 8:423. https://doi.org/10.3389/fphys.2017.00423 PMID: 28713281

91. Adami PE, Rocchi JE, Melke N, De Vito G, Bernardi M, Macaluso A. Physiological profile comparison between high intensity functional training, endurance and power athletes. Eur J Appl Physiol. 2022 Feb; 122(2):531–9. https://doi.org/10.1007/s00421-021-04858-3 PMID: 34853894

92. Adami PE, Rocchi JE, Melke N, Macaluso A. Physiological profile of high intensity functional training athletes. Journal of Human Sport & Exercise [Internet]. 2021 Jul [cited 2023 Feb 22]; 16(3):675–88. Available from: https://search.ebscohost.com/login.aspx?direct=true&db=s3h&AN=151677164&site=ehost-live.

93. Senefeld JW, Joyner MJ. Strength-Endurance Training Classes: Health Benefits and Injury Rates of an Emerging Cornerstone of Physical Activity. In: Mayo Clinic Proceedings. Elsevier; 2020. p. 437–9.

94. Makhlouf I, Castagna C, Manzi V, Laurencelle L, Behm DG, Chaouachi A. Effect of sequencing strength and endurance training in young male soccer players. The Journal of Strength & Conditioning Research. 2016; 30(3):841–50. https://doi.org/10.1519/JSC.0000000000001164 PMID: 26332782

95. Aspenes S, Kjendlie PL, Hoff J, Helgerud J. Combined strength and endurance training in competitive swimmers. Journal of sports science & medicine. 2009; 8(3):357. PMID: 24149998

96. Aagaard P, Andersen JL, Bennekou M, Larsson B, Olesen JL, Crameri R, et al. Effects of resistance training on endurance capacity and muscle fiber composition in young top-level cyclists. Scandinavian journal of medicine & science in sports. 2011; 21(6):e298–307. https://doi.org/10.1111/j.1600-0838.2010.01283.x PMID: 21362056

97. Hoffman JR, Fry AC, Howard R, Maresh CM, Kraemer WJ. Strength, speed and endurance changes during the course of a division I basketball season. The Journal of Strength & Conditioning Research. 1991; 5(3):144–9.

98. Chaabene H, Tabben M, Mkaouer B, Franchini E, Negra Y, Hammami M, et al. Amateur boxing: physical and physiological attributes. Sports medicine. 2015; 45:337–52. https://doi.org/10.1007/s40279-014-0274-7 PMID: 25358529

99. Franchini E, Del Vecchio FB, Matsushigue KA, Artioli GG. Physiological profiles of elite judo athletes. Sports medicine. 2011; 41:147–66. https://doi.org/10.2165/11538580-000000000-00000 PMID: 21244106

100. Gibala MJ, McGee SL. Metabolic adaptations to short-term high-intensity interval training: a little pain for a lot of gain? Exercise and sport sciences reviews. 2008; 36(2):58–63. https://doi.org/10.1097/JES.0b013e318168ec1f PMID: 18362686

101. Heinrich KM, Patel PM, O'Neal JL, Heinrich BS. High-intensity compared to moderate-intensity training for exercise initiation, enjoyment, adherence, and intentions: an intervention study. BMC public health. 2014; 14(1):1–6. https://doi.org/10.1186/1471-2458-14-789 PMID: 25086646

102. Armstrong N, Barker AR. Endurance training and elite young athletes. The elite young athlete. 2011; 56:59–83. https://doi.org/10.1159/000320633 PMID: 21178367

103. Martin BJ, Stager JM. Ventilatory endurance in athletes and non-athletes. Medicine and science in sports and exercise. 1981; 13(1):21–6. PMID: 7219131

104. Mastaloudis A, Leonard SW, Traber MG. Oxidative stress in athletes during extreme endurance exercise. Free Radical Biology and Medicine. 2001; 31(7):911–22. https://doi.org/10.1016/s0891-5849(01)00667-0 PMID: 11585710

105. Rønnestad BR, Mujika I. Optimizing strength training for running and cycling endurance performance: A review. Scandinavian journal of medicine & science in sports. 2014; 24(4):603–12. https://doi.org/10.1111/sms.12104 PMID: 23914932

106. Green ES, Williams ER, Feito Y, Jenkins NT. Physiological and Anthropometric Differences Among Endurance, Strength, and High-Intensity Functional Training Participants: A Cross-Sectional Study. Research Quarterly for Exercise and Sport [Internet]. 2023 Jan 2 [cited 2023 Jul 26]; 94(1):131–42. Available from: https://doi.org/10.1080/02701367.2021.1947468 PMID: 35302436

107. Gavanda S, Isenmann E, Geisler S, Faigenbaum A, Zinner C. The Effects of High-Intensity Functional Training Compared with Traditional Strength or Endurance Training on Physical Performance in Adolescents: A Randomized Controlled Trial. Journal of Strength and Conditioning Research. 2022 Mar 1; 36(3):624–32. https://doi.org/10.1519/JSC.0000000000004221 PMID: 35180184

108. Dawes J. Developing agility and quickness. Human Kinetics Publishers; 2019.

109. Deng N, Soh KG, Abdullah B, Huang D, Xiao W, Liu H. Effects of plyometric training on technical skill performance among athletes: A systematic review and meta-analysis. Plos one. 2023; 18(7): e0288340. https://doi.org/10.1371/journal.pone.0288340 PMID: 37459333

110. Kearns K. Agility for Fight Mobility. Black Belt [Internet]. 2010 Dec [cited 2023 Jan 6]; 48(12):81–3. Available from: https://search.ebscohost.com/login.aspx?direct=true&db=s3h&AN=59733416&site=ehost-live.

111. Sheppard JM, Young WB. Agility literature review: Classifications, training and testing. Journal of sports sciences. 2006; 24(9):919–32. https://doi.org/10.1080/02640410500457109 PMID: 16882626

112. Nimphius S, McGuigan MR, Newton RU. Relationship between strength, power, speed, and change of direction performance of female softball players. The Journal of Strength & Conditioning Research. 2010; 24(4):885–95. https://doi.org/10.1519/JSC.0b013e3181d4d41d PMID: 20300038

113. Pauole K, Madole K, Garhammer J, Lacourse M, Rozenek R. Reliability and validity of the T-test as a measure of agility, leg power, and leg speed in college-aged men and women. The Journal of Strength & Conditioning Research. 2000; 14(4):443–50.

114. Miller MG, Herniman JJ, Ricard MD, Cheatham CC, Michael TJ. The effects of a 6-week plyometric training program on agility. Journal of sports science & medicine. 2006; 5(3):459. PMID: 24353464

115. Sporis G, Jukic I, Milanovic L, Vucetic V. Reliability and factorial validity of agility tests for soccer players. The Journal of Strength & Conditioning Research. 2010; 24(3):679–86. https://doi.org/10.1519/JSC.0b013e3181c4d324 PMID: 20145571

116. Delextrat A, Grosgeorge B, Bieuzen F. Determinants of performance in a new test of planned agility for young elite basketball players. International journal of sports physiology and performance. 2015; 10 (2):160–5. https://doi.org/10.1123/ijspp.2014-0097 PMID: 24956606

117. Ouergui I, Franchini E, Messaoudi H, Chtourou H, Bouassida A, Bouhlel E, et al. Effects of Adding Small Combat Games to Regular Taekwondo Training on Physiological and Performance Outcomes in Male Young Athletes. Front Physiol. 2021; 12:646666. https://doi.org/10.3389/fphys.2021.646666 PMID: 33868014

118. DeBlauw J. High Intensity Functional Training Improves Flexibility in Overweight and Obese Adults: 654 Board #5 May 30 3:15 PM—5:15 PM. Medicine & Science in Sports & Exercise. 2018 May 2; 50:139–139.

119. Kemmochi M, Sasaki S, Ichimura S. Association between reduced trunk flexibility in children and lumbar stress fractures. Journal of orthopaedics. 2018; 15(1):122–7. https://doi.org/10.1016/j.jor.2018.01.014 PMID: 29657454

120. Chaabene H, Hachana Y, Franchini E, Mkaouer B, Chamari K. Physical and Physiological Profile of Elite Karate Athletes: Sports Medicine [Internet]. 2012 Oct [cited 2023 Feb 23]; 42(10):829–43. Available from: http://content.wkhealth.com/linkback/openurl?sid=WKPTLP:landingpage&an=00007256-201242100-00002 PMID: 22901041

121. Kaur J, Ganer N, Malik M, Kenkerwal G. CORRELATION OF SIT & REACH TEST AND INDIRECT INCLINOMETER MEASUREMENT OF HIP JOINT ANGLE IN HAMSTRING FLEXIBILITY TESTING IN INDIAN SCHOOL GOING GIRLS & BOYS. Romanian Journal of Physical Therapy/Revista Romana de Kinetoterapie. 2015; 21(35).

122. Chandler TJ, Kibler WB, Uhl TL, Wooten B, Kiser A, Stone E. Flexibility comparisons of junior elite tennis players to other athletes. The American journal of sports medicine. 1990; 18(2):134–6. https://doi.org/10.1177/036354659001800204 PMID: 2343979

123. Behm DG. The science and physiology of flexibility and stretching: implications and applications in sport performance and health. Routledge; 2018.

124. Gleim GW, McHugh MP. Flexibility and its effects on sports injury and performance. Sports medicine. 1997; 24:289–99. https://doi.org/10.2165/00007256-199724050-00001 PMID: 9368275

125. McNeal JR, Sands WA. Stretching for performance enhancement. Current sports medicine reports. 2006; 5:141–6. https://doi.org/10.1097/01.csmr.0000306304.25944.07 PMID: 16640950

126. Racil G, Jlid MC, Bouzid MS, Sioud R, Khalifa R, Amri M, et al. Effects of flexibility combined with plyometric exercises vs isolated plyometric or flexibility mode in adolescent male hurdlers. The Journal of Sports Medicine and Physical Fitness. 2019; 60(1):45–52. https://doi.org/10.23736/S0022-4707.19.09906-7 PMID: 31640314

127. Weerapong P, Hume PA, Kolt GS. Stretching: mechanisms and benefits for sport performance and injury prevention. Physical Therapy Reviews. 2004; 9(4):189–206.

128. Cosgrove SJ, Crawford DA, Heinrich KM. Multiple Fitness Improvements Found after 6-Months of High Intensity Functional Training. Sports [Internet]. 2019 Sep [cited 2022 Nov 28]; 7(9):203. Available from: https://www.mdpi.com/2075-4663/7/9/203 PMID: 31480686

129. Zagdsuren B, Evans GS, Inman C, Stone W, Arnett S, Schafer M, et al. Crossfit Vs. Circuit-training: Effects Of A Ten-week Training Program On Aerobic, Anaerobic And Flexibility Indicators.: 2924 Board# 239 May 29, 3: 30 PM-5: 00 PM. Medicine & Science in Sports & Exercise. 2015; 47 (5S):801.

130. Serafini PR, Feito Y, Mangine GT. Self-reported measures of strength and sport-specific skills distinguish ranking in an international online fitness competition. The Journal of Strength & Conditioning Research. 2018; 32(12):3474–84.

131. Proctor RW, Dutta A. Skill acquisition and human performance. Sage Publications, Inc; 1995.

132. Fellingham GW, Hinkle LJ, Hunter I. Importance of attack speed in volleyball. Journal of Quantitative analysis in Sports. 2013; 9(1):87–96.

133. Campos FAD, Bertuzzi R, Dourado AC, Santos VGF, Franchini E. Energy demands in taekwondo athletes during combat simulation. European journal of applied physiology. 2012; 112(4):1221–8. https://doi.org/10.1007/s00421-011-2071-4 PMID: 21769736

134. Sprey JW, Ferreira T, de Lima MV, Duarte A Jr, Jorge PB, Santili C. An epidemiological profile of CrossFit athletes in Brazil. Orthopaedic journal of sports medicine. 2016; 4(8):2325967116663706. https://doi.org/10.1177/2325967116663706 PMID: 27631016

135. Lee KYT, Hui-Chan CWY, Tsang WWN. The effects of practicing sitting Tai Chi on balance control and eye-hand coordination in the older adults: a randomized controlled trial. Disabil Rehabil. 2015; 37 (9):790–4. https://doi.org/10.3109/09638288.2014.942003 PMID: 25060039

136. Santana JC, Fukuda DH. Unconventional Methods, Techniques, and Equipment for Strength and Conditioning in Combat Sports. Strength Cond J [Internet]. 2011 Dec [cited 2022 Dec 29]; 33(6):64–70. Available from: https://www.webofscience.com/wos/woscc/summary/af85948b-a73c-43e4-a779-9451e6acf9ac-67c0f4c4/relevance/1.

137. Özgür E, BAYER R, BAYRAKDAROĞLU S. The Acute Effect of High-Intensity Functional Exercises on Circadian Rhythm and Anaerobic Performance Parameters. Gümüşhane Üniversitesi Sağlık Bilimleri Dergisi. 2022; 11(1):279–86.
